# Narrative exposure therapy for post-traumatic stress disorder and appetitive aggression among soldiers retiring from active service in Uganda

**DOI:** 10.21203/rs.3.rs-9091177/v1

**Published:** 2026-03-18

**Authors:** Dan Mwangye Bigirwa, Godfrey Zari Rukundo, Janet Nakigudde, Herbert Elvis Ainamani, Scholastic Ashaba

**Affiliations:** Mbarara University of Science and Technology; McMaster University; Makerere university; Kabale University; Mbarara University of Science and Technology

**Keywords:** Soldiers, Narrative exposure therapy, post-traumatic stress disorder, appetitive aggression, Uganda, sub-Saharan Africa

## Abstract

**Background:**

Narrative exposure therapy (NET) is a form of treatment for trauma disorders, particularly in individuals suffering from complex and multiple traumas. It is an evidence-based trauma-focused therapeutic approach designed to help individuals process and manage experiences of trauma, especially in the context of cumulative or complex trauma. Information on the effectiveness of narrative exposure therapy in managing post-traumatic stress disorder and appetitive aggression among retiring soldiers is limited. This study aimed to determine the effectiveness of narrative exposure therapy as a form of treatment for post-traumatic stress disorder (PTSD) and appetitive aggression (AA) among soldiers retiring from active service in Uganda.

**Methods:**

An interventional study was conducted among 70 retired Uganda People’s Defence Forces (UPDF) soldiers who had earlier been screened for post-traumatic stress disorder (PTSD) and appetitive aggression at Gaddafi Barracks in Jinja District, eastern Uganda, in preparation for retirement. At baseline, participants underwent narrative exposure therapy comprising eight 90-minute sessions delivered biweekly over four months. Sessions were conducted either at nearby barracks or within participants’ homes or community settings after retirement. Following completion of the four-month intervention period, participants were reassessed for PTSD and AA at endline.

**Results:**

Mean PTSD scores decreased from 19.96 (SD = 15.00) at baseline to 13.93 (SD = 8.96) at endline, representing a mean reduction of 6.04 points. This reduction was statistically significant, *t*(55) = 3.57, *p* < .001, indicating a significant decline in PTSD symptom severity following the intervention. Similarly, mean appetitive aggression scores decreased from 34.11 (SD = 6.59) at baseline to 10.39 (SD = 4.09) at endline, representing a mean reduction of 23.71 points. This reduction was statistically significant, *t*(58) = 20.29, *p* < .001, indicating a substantial decrease in appetitive aggression following narrative exposure therapy.

**Conclusion:**

Narrative exposure therapy demonstrated effectiveness in reducing symptoms of post-traumatic stress disorder and appetitive aggression among retiring soldiers in Uganda. These findings support consideration of integrating narrative exposure therapy into the UPDF retirement process prior to community reintegration.

## Introduction

Narrative exposure therapy (NET) is a form of treatment for trauma disorders particularly in individuals suffering from complex and multiple trauma (Elbert et al., 2022). It is an evidence-based, trauma-focused therapeutic approach designed to help individuals process and manage experiences of trauma, especially in the context of cumulative or complex trauma (Neuner et al., 2014; Robjant & Fazel, 2010). NET is an intervention to trauma-spectrum illnesses amongst survivors of several as well as complex trauma, built on the theory of the dual representation of traumatic memories (Neuner et al., 2008). It is thought to contextualize the particular associative elements of the fear network, the sensory, affective and cognitive memories of the trauma to understand and process the memory of a traumatic event in the course of the particular life of an individual (Neuner et al., 2014; Neuner et al., 2002; Shalev et al., 2024). It is noted that narrative exposure therapy permits reflection on the person’s entire life as a whole, nurturing a sense of personal identity (Robjant & Fazel, 2010). In narrative exposure therapy sessions, the formerly unrelated fear network for the traumatic events is stimulated and connected with the cold memory, so as to contextualize the events (Neuner et al., 2014). This is aimed at completing an autobiographical memory by connecting the hot and cold memory in this way, contextualizing each event (Neuner et al., 2014). Furthermore, the fear reaction can be reversed through exposure to the traumatic memory (Jongedijk, 2014).

Post-traumatic stress disorder (PTSD) is one of the commonest mental health challenges among veterans and service members (Armenta et al., 2018; Inoue et al., 2021). The risk of PTSD is highest among military personnel who are deployed in war zones due to exposure to various traumatic events (Obuobi-Donkor et al., 2022) and most especially among the soldiers who get injured in their line of duty (Marmar et al., 2015). The prevalence of PTSD among military personnel with a history of combat exposure has been found to range from 10% to 15%, with a lifetime prevalence of up to 30% (Donoho et al., 2017; Hofmann et al., 2003; Trautmann et al., 2017). Posttraumatic stress disorder (PTSD) consists of a cluster of symptoms that severely impact daily functioning and quality of life (Balayan et al., 2014; Giacco et al., 2013). The psychological and behavioral symptoms include re-experiencing of the trauma, avoidance of stimuli associated with the trauma, negative alterations in cognitions and mood and hyperarousal (American Psychiatric Association, 2005). PTSD can have far-reaching consequences significantly affecting service members and their families (Armenta et al., 2018; Tanielian, 2008). While the course of PTSD among the veterans varies, it has been noted to be persistent and may last a lifetime (Armenta et al., 2018; Marmar et al., 2015).

In addition to PTSD, soldiers are subjected to a variety of severe forms of violence, including gang-related violence, emotional abuse, harassment, combat-related injuries, torture, and fatalities (Robjant et al., 2020). Exposure to violence including organized violence increases the risk of mental disorders such as trauma-related illnesses, depression, substance abuse, and appetitive aggression (Baez et al., 2019). When armed groups are exposed to high levels of violence during combat, their emotions change from fear to exhilaration and arousal which can result in appetitive aggression (Hermenau et al., 2013). Appetitive aggression is a form of hostile behavior that is driven by the purpose of gaining a social status, as well as the self-rewarding desire and pleasure of perpetrating pain through violence (Elbert et al., 2010; Hecker et al., 2012; Weierstall et al., 2013).

The Ugandan military has been involved in various operations both domestically and in neighboring countries including a thirteen year war insurgency in northern Uganda with reports of human rights violations by soldiers including killings, rapes, and physical assault (Minch, 2011). The military’s role in maintaining security can sometimes lead to tensions and violence, especially in conflict-prone areas (Namwase, 2020; Sheehan, 2010). Many of these wars have been reported to be traumatic in nature, involving exposure to near-fatal experiences (Dami et al., 2018). Some soldiers have demonstrated hostile behavior in the community after their time in the army including the killing of fellow citizens (MacManus et al., 2015). As such, some soldiers have exhibited aggressive behavior after returning to the community. Therefore, this study aimed at determining the role of narrative exposure therapy as a treatment strategy among soldiers with post-traumatic stress disorder and appetitive aggression retiring from active service in Uganda.

## Methods

### 2.1. Study design

The study was quasi-experimental in nature, employing a one-group pretest-posttest research design in which the outcome of interest was measured at two-time intervals i.e., before and after exposing a non-random group of participants to an intervention/treatment. Participants in this quasi-experimental study were part of the 247 soldiers retiring from active service who were recruited between July and August 2023. Initially all the 247 participants were screened for PTSD, AA, depression, substance abuse disorder, childhood trauma, and alcohol use disorder PTSD and appetitive aggression. The 70 participants who took part in the intervention were those who screened positive for PTSD and AA. Of the 70 participants, 11 had both AA and PTSD, 21 had PTSD signs and symptoms only and 38 had AA signs and symptoms only. Participants with PTSD and AA underwent narrative exposure therapy as the intervention

### 2.2. Screening setting

The screening was conducted in the Gaddafi military barracks in Jinja district, eastern Uganda where soldiers retiring from the army in Uganda usually assemble for one month before being retired and integrated into the community. Gaddafi military barracks is home to over 4000 soldiers and each year receives several soldiers ranging from 400–700 for retirement preparation. Gaddafi military barracks is located approximately 87 kilometers east of Kampala the capital city of Uganda (Fecitt, 2008). It is the oldest military barracks in Uganda, established during the colonial rule in Uganda. It was the home of the first battalion of King African rifle (KAR) and headquarter for the army during colonial rule and after independence up to 1964 (Fecitt, 2008).

### 2.3 Sample size

The sample size was based on the baseline screening results of those who had signs and symptoms of PTSD and appetitive aggression, a total of 70 participants were enrolled for intervention.

### 2.4 Sampling procedure

The screening procedure employed a stratified random sampling design. Soldiers residing in the barracks were first stratified into eight groups based on length of service, rank (officers and militants), and position held at the time of retirement (command or administrative roles). From these strata, eligibility was further determined by combat exposure. Participants who held administrative positions at retirement were included only if they had previously engaged in combat operations, while those without any combat experience were excluded. Similarly, individuals who occupied command positions at the time of retirement and met the inclusion criteria were also considered eligible for participation. The sampling frame comprised all individuals within the retiring cohort who satisfied the predefined inclusion criteria, while those outside the retiring group or failing to meet eligibility requirements were excluded. This approach ensured adequate representation across relevant service characteristics while maintaining consistency with the study’s objectives.

### 2.5 Recruitment procedures

Participants were recruited from retiring personnel of the Uganda People’s Defence Forces (UPDF) who were undergoing routine retirement-related screening. During this process, potential participants were screened at baseline for post-traumatic stress disorder (PTSD) and appetitive aggression (AA). Individuals who screened positive for both conditions were considered eligible for enrolment into the intervention phase of the study. At baseline, all eligible participants underwent a comprehensive assessment that included measures of PTSD, AA, depression, childhood trauma exposure, substance use disorder, and alcohol use disorder. Those who met the screening criteria for PTSD and AA and who provided informed consent were subsequently enrolled to receive NET. Recruitment was conducted on a rolling basis until the desired sample size was achieved. Participants were informed about the nature of the intervention, the duration and frequency of therapy sessions, and their right to decline or withdraw from the study at any time without consequence. Individuals identified as eligible and willing to participate were scheduled for intervention sessions following baseline assessment. This recruitment approach ensured that the intervention was offered to individuals with demonstrated clinical need while integrating seamlessly into existing retirement and health screening processes within the UPDF.

#### Inclusion criteria

Participants were eligible for participation if they had served in the army for at least 10 years, had participated in at least one combat engagement, and provided written informed consent to participate in the study. The threshold of 10 years of service was selected to minimize the inclusion of individuals who retired prematurely on voluntary or medical grounds before completing a substantial period of military service. Service duration of ten years or more was therefore considered sufficient to reflect meaningful military engagement and to ensure inclusion of personnel who had served long enough to approach standard retirement pathways (Fecitt, 2008).

#### Exclusion criteria

Potential participants were excluded if they were intoxicated or exhibited overt symptoms of active mental illness at the time of the interview. These symptoms included marked mood instability, unusual or disorganized behavior, incoherent or uncoordinated speech, disorientation, apathy, social withdrawal, heightened sensitivity, or observable aggression. Such conditions were considered likely to impair participants’ capacity to adequately comprehend the study information, understand the questionnaire items, and provide valid written informed consent

### 2.6 Data collection

Data were collected by trained research assistants using semi-structured interviews and standardized questionnaires. The research assistants were qualified counselling psychologists working within the UPDF and participated in the study with authorization from their respective departments. Prior to data collection, research assistants received a one-day training from the principal investigator on the standardized administration of study instruments, ethical research conduct, and appropriate techniques for addressing sensitive questions. Interviews were conducted in private settings to ensure confidentiality and promote open disclosure, with each assessment taking approximately 45–60 minutes to complete.

### 2.7 Measures

All screening instruments were compiled into a single questionnaire that was translated from English into Kiswahili and back translated into English to ensure fidelity. Kiswahili language is an official military language in Uganda used in all their commands and is seconded by English. Interviews were carried in the classrooms and the dining hall where privacy was ensured to enable participants freely respond to questions without interference. The questionnaire included social demographic factors such as sex, age, rank, level of education, length of service in the army and marital status, PTSD checklist, Hopkins symptoms checklist (HSCL-25), the life events checklist for DSM-V, alcohol use disorder identification test, drug abuse screening test and the childhood trauma questionnaire.

#### Appetitive aggression scale

Appetitive aggression was assessed using the appetitive aggression scale which is a 15-item scale that was developed using more than 2000 numbers drawn from diverse conflict affected areas showing and excellent reliability with a Cronbach’s alpha of 0.85 (Weierstall & Elbert, 2011). Examples of the items in the appetitive aggression scale include “Is it exciting for you if you make an opponent really suffer*?*” “Once fighting has started do you get carried away by the violence?”. The appetitive aggression is scored on a 5-point Likert scale ranging from 0 (totally disagree) to 4 (totally agree). Items are summed up with minimum score of 0 and a maximum score of 60 and higher scores indicate appetitive depression. It has been used in Uganda before among former child soldiers in northern Uganda with Cronbach’s alpha of 0.85 (Weierstall & Elbert, 2011). The scale had a Cronbach alpha of 0.79 in this study.

#### The Hopkins symptoms checklist

The Hopkins Symptoms Checklist (HSCL-25) is a self-report symptom inventory (Derogatis et al., 1974; Skogen et al., 2017). The 25-item HSCL was derived from the 90-item Symptom Checklist (SCL-90) and is usually administered as a 15-item depression subscale and a 10-item anxiety subscale (Derogatis & Unger, 2010). Participants are asked to rate the frequency of each symptom in the last seven days on a four-point Likert-type scale, from “not at all” (1) to “very much” (4). The average score of 1.75 or higher suggests significant depressive symptoms (Derogatis & Unger, 2010). The HSCL-25 has been validated for use in Uganda with good psychometric properties demonstrated by a Cronbach’s alpha of 0.91 (Ashaba et al., 2018). The HSCL-25 had a Cronbach’s alpha of 0.92 in this study.

#### The Life Events Checklist for DSM-V

The Life Events Checklist for DSM-5 (LEC-5) is a self-report measure intended to screen for likely traumatic events in a person’s lifetime (Weathers et al., 2013). The Life Events Checklist for DSM-5 (LEC-5) assesses exposure to a variety of potentially traumatic events which include natural disasters like floods, fire or explosion, transportation accidents, serious accidents at work or home, exposure to toxic substances, physical assault or assault with a weapon, sexual assault, combat exposure, captivity, life-threatening illness or injury, and serious injury (Weathers et al., 2013). The Life Events Checklist for DSM-5 has been used in Kenya among adults with potential traumatic events (Kwobah et al., 2022). While the LEC-5 does not provide a total score it helps to identify whether the person has experienced one or more traumatic events. This information can be used in combination with other assessments to determine if the events meet the criteria for PTSD or other trauma-related conditions (Rzeszutek et al., 2018). The LEC-5 had a Cronbach’s alpha of 0.84 in this study.

#### Alcohol use disorder identification test

Alcohol use disorder was assessed using the alcohol use disorder identification test (AUDIT) questionnaire which assesses for alcohol use problems, alcohol use behaviors, and dependence (Saunders et al., 1993). It is a ten item questionnaire scored on a five point Likert scale ranging from 0–4 with options of never (=0) , monthly or less (=1), 2–3 times a month (=2), 2–4 times a week (=3), and 4 or more times a week (=5) (Babor et al., 2001; Reinert & Allen, 2007). The AUDIT is unique among alcohol related screening instruments in that it is designed to measure a range of risk levels from low-risk drinking to hazardous drinking and alcohol use disorders (de Meneses-Gaya et al., 2009; Reinert & Allen, 2007). Low risk drinking denotes alcohol consumption in very small amounts which is also known as safe drinking/social drinking, while hazardous drinking is a pattern of alcohol consumption that is associated with alcohol related problems although it does not meet the minimum criteria for alcohol use disorder (de Meneses-Gaya et al., 2009; De Silva et al., 2008). The AUDIT has been documented as a reliable and valid measure in identifying alcohol use disorder, hazardous consumption and harmful alcohol use and it has also been found to be a valid indicator for severity of alcohol dependence (Källmén et al., 2019). The audit had a Cronbach’s alpha of 0.84 in this study.

#### Drug abuse screening test

Drug abuse was assessed using the Drug Abuse screening Test (DAST) which is a 10-item scale score on yes and no responses (Scherer et al., 2013). It’s used to assess the use of drugs not including alcohol or tobacco use in the past 12 months and is easy to administer (Scherer et al., 2013; Yudko et al., 2007). The DAST is a biologically validated instrument that is easy to use and may have valuable implications as a research tool among both clinical and non-clinical populations (Scherer et al., 2013). The DAST has previously been used among University students in Uganda (Kaggwa et al., 2022). The DAST has been evaluated good psychometric properties with a Cronbach’s ranging from 0.74 to 0.94 across different studies (Shirinbayan et al., 2020; Yudko et al., 2007). The DAST had a Cronbach alpha of 0.763 this study.

#### Childhood trauma questionnaire

Childhood trauma was assessed using the28-item childhood Trauma questionnaire (CTQ). The CTQ consists of 28 items and 5 subscales that physical, emotional, and sexual abuse; and emotional and physical neglect (Bernstein, 1998). Items 10, 16, and 22 comprise the denial subscale. Each item is measured on a five-point Likert scale ranging from “Never true” =0 to “Very often true =3 (Bernstein et al., 2003). The CTQ items are elicited in context of “when I was growing up” which is followed by the description of a traumatic event that they could have experienced. Sample items include: “When I was growing up, I got hit so hard by someone in my family that I had to see a doctor or go to the hospital”; and “When I was growing up, people in my family said hurtful or insulting things to me. The cut-off scores for different subscales are ≥6 for sexual abuse; ≥8 for physical abuse; ≥9 for emotional abuse; ≥8 for physical neglect; and≥10 emotional neglect (Fink et al., 1995). We dichotomized the subscale scores at the specified cut-offs, so that participants who scored above the cut-off were classified as having experienced abuse or neglect, while those who scored lower were classified as not having experienced abuse or neglect. The CTQ has been used among adolescents living with HIV in Uganda where it had a Cronbach’s alpha of 0.86 (Ashaba et al., 2021). The scale had a Cronbach alpha of 0.72 in this study.

#### The Post traumatic stress disorder (PTSD) checklist

The PTSD Checklist for DSM-5 (PCL-5) is a 20- self-report measure that assesses the presence and severity of PTSD symptoms (Bovin et al., 2016; Wortmann et al., 2016). Items on the PCL-5 correspond with DSM-5 criteria for PTSD. The PCL-5 is constructed on a five Likert scale rated as not at all =1, a little a bit =2, moderately=3, quite a bit =4 and extremely =5 responses (Blevins et al., 2015; Bovin et al., 2016). The score on the PCL-5 is done by summing up all item scores and a total score of 31–33 or higher suggests the presence of PTSD (Blevins et al., 2015; Forkus et al., 2023). The PCL-5, aligns with the DSM-5 criteria for PTSD and has shown strong validity and reliability across various settings (Forkus et al., 2023). The PCL-5 has been validated among University students in Rwanda with good psychometric properties (Cronbach’s alpha = 0.85) (Niyonsenga et al., 2021) and has also been used in eastern Uganda among landslide survivors where it demonstrated a Cronbach’s 0.84 (Kabunga et al., 2022). The scale had as Cronbach’s alpha of 0.86 in this study.

### 2.8 Narrative exposure therapy

Narrative exposure therapy (NET) was delivered over eight sessions, each lasting approximately 60–90 minutes. The first session focused on psychoeducation regarding trauma, PTSD, and appetitive aggression. During the second session, participants constructed a lifeline, symbolized by a rope with a coiled end; stones represented traumatic experiences, while flowers symbolized positive life events, arranged in chronological sequence. The third session involved the initiation of the life narrative, beginning at birth and progressing to the first traumatic event. The fourth and subsequent sessions focused on rereading the narratives documented in earlier sessions while continuing the narration of subsequent life events, including traumatic experiences and acts associated with appetitive aggression. The final session involved a complete rereading of the narrative, followed by participant verification and signing of the document. At endline, participants spent approximately 40–45 minutes completing semi-structured interviews designed to assess perceived changes in post-traumatic stress disorder symptoms and appetitive aggression following the NET intervention.

### 2.9 Data analysis

Data were analyzed using Stata version 14. Categorical variables were summarized using frequencies and percentages, while continuous variables were summarized using means and standard deviations. The effect of the intervention was assessed by using a paired t- test to compare participants score on PTSD and AA before and after the intervention. Statistical significance was determined using a p-value of less than 0.05, with corresponding confidence intervals reported.

### 2.10 Ethical considerations

We received ethical approval from the Mbarara University research ethics committee (MUST-2023–823) and the study was registered and cleared by the Uganda National for Science and Technology (HS2949ES). Administrative clearance was sought from the Uganda chief of defense forces through joint staff human resource and the chief joint staff as well as the head of retirement exercise at Gaddaffi military barracks in Jinja to do baseline screening. Participants provided written informed consent before they were enrolled into baseline screening. Each participant received five thousand Ugandan shillings as compensation for their time taken to participate in the baseline screening. At baseline participants who were found with signs and symptoms of PTSD and appetitive aggression were called on phone which they provided and arrangements were made to have them undergo treatment in private rooms to ensure confidentiality. After the period of four months, they consented again to fill in the interview guide to get the outcome results.

## Results

### 3.1 Sample characteristics

The sample consisted of 70 participants with a mean age of 44.9 years (SD = 7.81) and a mean length of service of 21.8 years (SD = 8.22). The majority were male (65, 93%) with 5 females (7%). Regarding education, 29 (41%) had primary education, 21 (30%) had lower secondary, and 20 (29%) had upper secondary education or higher. In terms of marital status, 65 (73%) were married and 5 (7%) were single. By rank, 8 (11%) were commissioned officers, 30 (43%) senior non-commissioned officers, and 32 (46%) junior non-commissioned officers (Table 1).

### 3.2 The effectiveness of Narrative exposure therapy in the management of PTSD and AA among Ugandan soldiers retiring from active service.

As shown in Table 2, The mean PTSD score declined from 19.96 (SD = 15.00) at baseline to 13.93 (SD = 8.96) at endline, corresponding to a mean reduction of 6.04 points. This reduction was statistically significant, *t*(55) = 3.57, *p* < .001, indicating a meaningful decrease in PTSD symptom severity following the intervention. Similarly, the mean score for appetitive aggression decreased substantially from 34.11 (SD = 6.59) at baseline to 10.39 (SD = 4.09) at endline, yielding a mean reduction of 23.71 points. This change was also statistically significant, *t* (58) = 20.29, *p* < .001, suggesting that Narrative Exposure Therapy (NET) was highly effective in reducing levels of appetitive aggression among the participants.

## Discussion

Following the implementation of Narrative Exposure Therapy (NET), participants demonstrated meaningful reductions in both post-traumatic stress disorder (PTSD) symptoms and appetitive aggression from baseline to endline. Levels of PTSD symptom severity and appetitive aggression were consistently lower after completion of the intervention, suggesting that NET was associated with improved mental health outcomes among this group of retiring soldiers. Importantly, to our knowledge, this study represents the first evaluation in Uganda of the effectiveness of NET for addressing both PTSD and appetitive aggression among military personnel, and specifically among individuals transitioning out of active service. These findings contribute novel evidence to the limited body of trauma-focused intervention research within military populations in low-resource settings.

The findings of this study are consistent with a growing body of evidence demonstrating the effectiveness of Narrative Exposure Therapy (NET) in reducing both post-traumatic stress disorder (PTSD) symptoms and appetitive aggression among trauma-exposed populations. Previous studies have shown NET to be highly effective in addressing PTSD and aggressive tendencies that develop in the context of repeated and severe trauma (Hecker et al., 2015). In particular, the application of NET among former child combatants and ex-soldiers has been associated with reductions in fear-based and aggressive behaviour, while also supporting psychological recovery and social reintegration in post-conflict settings (Hermenau et al., 2013). NET has also demonstrated effectiveness among survivors of planned violence and extreme human rights violations, including torture and prolonged persecution (Hensel-Dittmann et al., 2011; Neuner et al., 2011). Its utility has further been documented among individuals with complex trauma histories, including war survivors and refugees, where NET has been shown to reduce trauma-related symptoms and improve overall mental health functioning (Jongedijk, 2014; Mauritz et al., 2016; Schauer & Elbert, 2015; Steuwe et al., 2021). Importantly, NET has also been applied within forensic and offender rehabilitation settings, where it has been effective in addressing the co-occurrence of traumatic victimization, PTSD symptoms, and appetitive aggression linked to repeated exposure to violence (Köbach, 2015). Evidence from Northern Uganda further supports these findings, with studies among former child soldiers showing greater reductions in PTSD symptoms following NET, underscoring its relevance for conflict-affected populations in similar contexts (Hecker et al., 2015). Existing literature indicates that NET is both feasible and effective in addressing trauma spectrum disorders among individuals exposed to multiple and complex traumatic events, particularly in low- and middle-income and post-conflict settings. The current study extends this evidence by demonstrating comparable benefits among retiring military personnel in Uganda, reinforcing the potential role of NET in reducing trauma-related symptoms and aggressive behaviour during the transition from military to civilian life (Neuner et al., 2014; Robjant et al., 2019).

Several limitations should be considered when interpreting the findings of this study. First, the study population was restricted to soldiers who had recently retired from active service in Uganda. As a result, the findings may not be directly generalizable to military personnel who remain in active service, whose experiences, exposures, and support systems may differ.

Second, the study employed a pre–post intervention design without a control group. While this design allowed for the assessment of changes over time, it limits the ability to attribute observed improvements solely to the intervention. Other factors occurring alongside the intervention such as natural recovery, changes in life circumstances following retirement, or external support may also have influenced the outcomes. Consequently, the effects observed in this study should be interpreted with caution, and future research using controlled designs would be valuable in strengthening causal inferences. Despite these limitations, the study has several important strengths. To our knowledge, this is the first study in Uganda to evaluate the effectiveness of Narrative Exposure Therapy for both post-traumatic stress disorder and appetitive aggression among retiring military personnel, addressing a significant gap in the literature. The study focused on a clinically relevant and underserved population undergoing a critical life transition, a period during which mental health needs are often heightened. In addition, the intervention was delivered using a structured, evidence-based therapeutic approach by trained personnel, enhancing the internal validity of the findings. The use of standardized and validated assessment tools further strengthens confidence in the observed changes. These strengths support the relevance of the findings and provide a strong foundation for future controlled studies and for the potential integration of trauma-focused interventions within military and veteran mental health services in Uganda.

## Conclusion

This study provides evidence that Narrative NET is an effective intervention for reducing post-traumatic stress disorder symptoms, appetitive aggression, and depressive symptoms among retired soldiers in Uganda. The structured, trauma-focused nature of NET enables individuals to safely recount, process, and integrate traumatic experiences within a supportive therapeutic environment. Through this process, participants are better able to make sense of their experiences, leading to reduced psychological distress and improved emotional well-being. Beyond individual symptom reduction, the observed improvements suggest that NET may also support smoother social and psychological reintegration of retired soldiers as they transition from military to civilian life. Given the high burden of trauma exposure among military personnel, these findings highlight the potential value of integrating NET into the UPDF retirement and post-service support framework, particularly for individuals with significant trauma histories. Incorporating trauma-focused interventions such as NET at the point of retirement may help address unmet mental health needs and promote longer-term recovery and community reintegration.

## Figures and Tables

**Figure 1:**
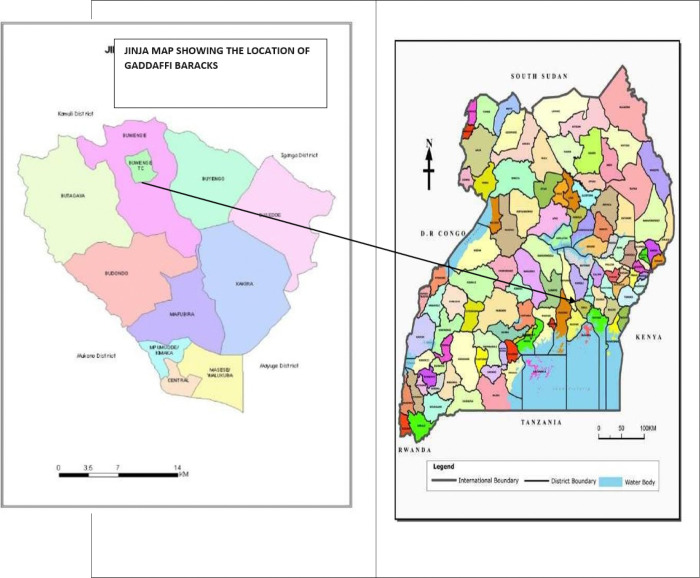
Unnumbered image in Methods section.

**Table 1.** Descriptive statistics of the sociodemographic characteristics of the participants (n=70)

Characteristic	N or mean (SD)	%
Age (Mean/SD)	44.9 (7.81)	
Length of Service (Mean/SD)	21.8 (8.22)	
**Sex**		
Male	65	93
Female	5	7
**Level of Education**		
Primary	29	41
Lower Secondary	21	30
Upper Secondary and above	20	29
**Marital Status**		
Married	65	73
Single	5	7
**Rank**		
Commissioned Officers	8	11
Senior Non-Commissioned officers	30	43
Junior Non-Commissioned officers	32	46
**Audit Categories**		
Low risk	55	79
Moderate to Hazardous	15	21
**DAST Categories **		
Low Levels	63	90
Optimal Substantial	5	7
Substance use problem	2	3
**Depression Categories**		
Not Depressed	49	70
Depressed	21	30

Note: n-Frequency

**Table 2.** Results of a paired sample t-test comparing pre and post mean scores of PTSD and Appetitive Aggression among retiring soldiers following the Narrative Exposure Therapy intervention

Variable	Pre-NEC	Post-NEC		
	M(SD)	M(SD)	*t(*df)	P
PTSD, Total Score	19.96(15)	13.93(8.96)	3.57(55)	<0.001
Appetitive Aggression, Total Score	34.11(6.59)	10.39(4.09)	20.29(55)	<0.001

**Note:** M-Mean, SD- Standard deviation, *t* – test statistic, df- degrees of freedom

## Data Availability

The datasets generated and/or analyzed during the current study are not publicly available due to research ethics board restrictions but are available from the corresponding author upon reasonable request.
